# Insoluble glycogen, a metabolizable internal adsorbent, decreases the lethality of endotoxin shock in rats

**DOI:** 10.1080/09629359791442

**Published:** 1997-12

**Authors:** S. Sipka, G. Bot, P. Gergely, L. Bertók, J. Csongor, P. Sápy, M. Szappanos, J. Nemes, E. Duda, G. Szegedi

**Affiliations:** 1 3rd Department of Internal Medicine University Medical School of Debrecen Hungary; 2 Department of Medical Chemistry University Medical School of Debrecen Hungary; 3 Central Isotope Laboratory University Medical School of Debrecen Hungary; 4 2nd Department of Surgery University Medical School of Debrecen Hungary; 5 Department of Microbiology University Medical School of Debrecen Hungary; 6 National Research Institute for Radiology Budapest Hungary; 7 Biological Research Institute of Hungarian Academy of Sciences Szeged Hungary

## Abstract

Insoluble glycogen is an enzymatically modified form of naturally occurring soluble glycogen with a great adsorbing capacity. It can be metabolized by phagocytes to glucose. In this study we used insoluble glycogen intravenously in the experimental endotoxin shock of rats. Wistar male rats were sensitized to endotoxin by Pb acetate. The survival of rats were compared in groups of animals endotoxin shock treated and non-treated with insoluble glycogen. Furthermore, we have determined *in vitro* the binding capacity of insoluble glycogen for endotoxin, tumour necrosis factor alpha, interleukin-1 and secretable phospholipase A_2_. Use of 10 mg/kg dose of insoluble glycogen could completely prevent the lethality of shock induced by LD_50_ quantity of endotoxin in rats. All animals treated survived. Insoluble glycogen is a form of ‘metabolizable internal adsorbents’. It can potentially be used for treatment of septic shock.


Mediators of Inflammation, 6, 319–322 (1997)

### Insoluble glycogen, a metabolizable internal adsorbent, decreases the lethality of endotoxin shock in rats

S. Sipka[1,CA], G. Bot[2], P. Gergely[2], L. Bertók[6], J. Csongor[3], P. Sápy[4], M. Szappanos[4], J. Nemes[5], E. Duda[7] and G. Szegedi[1]

[1]3rd Department of Internal Medicine, [2]Department of Medical Chemistry, [3]Central Isotope Laboratory, [4]2nd Department of Surgery, [5]Department of Microbiology, University Medical School of Debrecen; [6]National Research Institute for Radiology, Budapest; [7]Biological Research Institute of Hungarian Academy of Sciences, Szeged, Hungary

[CA]Corresponding Author
Tel/Fax: (+36) 52 414 969

**Insoluble glycogen is an enzymatically modified form of naturally occurring soluble glycogen with a great adsorbing capacity. It can be metabolized by phagocytes to glucose. In this study we used insoluble glycogen intravenously in the experimental endotoxin shock of rats. Wistar male rats were sensitized to endotoxin by Pb acetate. The survival of rats were compared in groups of animals endotoxin shock treated and non-treated with insoluble glycogen. Furthermore, we have determined *in vitro* the binding capacity of insoluble glycogen for endotoxin, tumour necrosis factor alpha, interleukin-1 and secretable phospholipase $A_2$. Use of 10 mg/kg dose of insoluble glycogen could completely prevent the lethality of shock induced by $LD_{50}$ quantity of endotoxin in rats. All animals treated survived. Insoluble glycogen is a form of 'metabolizable internal adsorbents'. It can potentially be used for treatment of septic shock.**

**Key words:** Glycogen, Tumour necrosis factor, Interleukin-1, Phospholipase $A_2$, Platelet activating factor, Endotoxin shock

### Introduction

In the pathogenesis of endotoxin or septic shocks the main roles are played by secondary mediators, e.g. tumour necrosis factor alpha (TNF-$\alpha$), interleukin-1 (IL-1), phospholipase $A_2$ ($PLA_2$) and platelet activating factor (PAF).[1–5] These substances cause generalized lesions of capillaries resulting in hypotonia with the symptoms of concomitant multiple organ dysfunction syndrome.[1] Numerous biologically potent materials, toxins, cytokines, enzymes, lipid and peptide mediators can be simultaneously present in the circulation during septic shock. Blocking only one of these mediators cannot be enough.[6,7] Besides extracorporeal adsorbents,[8,9] intravascular adsorbents like kaolin, aluminium and magnesium silicate were found to have beneficial, simultaneously detoxificating effects in animal experiments.[10] However, at a certain level, they were also toxic. Therefore, there is a need to develop drugs regarded as 'metabolizable internal adsorbents'.

Insoluble glycogen (ISG) is an enzymatically modified new form of naturally occurring glycogen. In consequence of its enlarged surface, it has an increased adsorbent capacity compared with natural glycogen or amylum. Phagocytes can engulf ISG and degrade it to glucose in the lysosomes, helping the metabolism of phagocytosing cells. Insoluble glycogen represents a potent adsorbing capacity in blood. In phagocytes it helps the metabolism during the biological neutralization of toxic materials. It is a 'metabolizable internal adsorbent' able to decrease the lethality in endotoxin (*Escherichia coli* lipopolysaccharide) induced shock of rats.

### Materials and Methods

### Preparation of insoluble glycogen

Insoluble glycogen was prepared and made by our method in our laboratory.[11] Briefly, the enzymatic elongation of natural, soluble glycogen took place by phosphorylase-a and glucose 1 phosphate, resulting in a new, artificial form, called insoluble glycogen (ISG) forming insoluble particles with 1 mm average diameter in water or buffer milieu. Sterile and endotoxin-free dishes and devices were used throughout the preparation. ISG represents an enlarged surface for aspecific and simultaneous adsorption of various molecules in a solution. The suspensions of insoluble glycogen were prepared for intravenous treatment: 20 mg/ml of ISG was used in sterile and endotoxin-free phosphated buffer in saline (PBS).

© 1997 Rapid Science Publishers 

### Rat model of endotoxin shock[12]

Wistar male rats (200 g) were presensitized to endotoxin (*E. coli* lipopolysaccharide) by intravenous (i.v.) injection of 5 mg, endotoxin free Pb (lead) acetate (Reanal, Hungary). The $LD_{50}$ dose of i.v. *E. coli* lipopolysaccharide (LPS, endotoxin, ET) was found to be 2 μg, whereas the $LD_{100}$ dose of LPS was 5 μg. Ten mg/kg of i.v. insoluble glycogen was applied just before LPS. ISG, Pb acetate, LPS were injected i.v. through the same needle but from various syringes in the sequence: ISG, Pb acetate, LPS. The lethality and survival were evaluated after 12 h. The results were calculated on the basis of three separate experiments.

The experimental protocol was approved by the Local Animal Experimental Ethical Committee in 1996.

### Binding assays

$^{99m}$Tc labelling of *E. coli* LPS (endotoxin), $^{125}$I labelling of human phopholipase $A_2$ (14 kD purified in our laboratory), rabbit phosphorylase-a (purified in our laboratory), human recombinant TNF-α (prepared by Duda *et al.*), human IgG (Human Serobacteriological Institute, Hungary) were carried out in our laboratories. The C3b activating and binding capabilities of ISG was measured in citrated rabbit blood by determination of the amount of C3b consumed during the activation of alternative way of complement system. The *in vitro* binding capacity of 1 mg of ISG was calculated for various substances and the masses of bound materials were expressed in mg of bound molecules/1 mg of ISG. We have preferred to use this form of calculation instead of counting in mols, because this formula reflects better the relations of quantities to each other. For example: 0.1 mg of phosphorylase-a represents $10^{-9}$M, likewise 0.015 mg of phospholipase $A_2$, showing that the expression of the masses of bound molecules in mg and not in M can describe better the real binding situations on the surfaces of ISG particles. The results represent the average values of two separate binding measurements with duplicated samples ($n = 4$).

### Measurement of chemiluminescence (CL)

0·5 ml of heparinized rat and human blood samples were used in diluted forms by phosphate buffered saline (PBS) and stimulated by 0·5 mg/ml of zymosan (Mannozym, Human Serobacteriological Institute, Hungary), ISG and ISG opsonized with rabbit phosphorylase-a. The CL, the number of photons emitted by phagocytes were measured in $10^{-7}$ M luminol (5-amino, 2,3-dihydro, 1,4-phtalazinedione, Sigma, USA) milieu by a Nuclear Chicago Isocap/300 liquid scintillation counter (Searle Industries, USA) in the off coincidence mode.[13]

### Results

The $LD_{50}$ dose of *E. coli* endotoxin (ET, lipopolysaccharide, LPS) was found to be 2 μg in rats sensitized to endotoxin by Pb (lead) acetate. We have found that 10 mg/kg dose of ISG given intravenously, simultaneously with Pb acetate and ET (by the same needle but from different syringes in the series: ISG, Pb acetate and LPS) could completely prevent the lethal effect of $LD_{50}$ dose of ET. In this group, all 12 animals survived (Table 1).

In the pathomechanism of endotoxin shock, the effects of circulating ET, TNF, IL-1, $PLA_2$, PAF are regarded to be the main causative factors. For the explanation of the *in vivo* protective effect of ISG in the rat model of endotoxin shock, we have measured its binding capacity of these materials *in vitro*. The aim of the binding assays was to determine the amount of these biologically important molecules bound by ISG aspecifically, by adsorption, in order also to prove the possibility of the occurrence of the phenomenon *in vivo*. Table 2 shows that ISG could bind ET labelled with $^{99m}$Tc dose dependently, *in vitro*.

Insoluble glycogen was found to bind $^{125}$I-labelled human recombinant TNF-α, IL-1, human secretable $PLA_2$ and human IgG (Table 3). The greatest binding capacity of ISG was found on phosphorylase-a, it was still rather high on IgG, C3b and phospholipase $A_2$, whereas on the cytokines, TNF-α and IL-1, it was rather low (Table 3).

Table 4 shows that the luminol amplified chemiluminescence of phagocytes. ISG, like zymosan, increased the chemiluminescence in the samples of both human and rat bloods. However, when phosphorylase-a and the ISG

**Table 1**. Protecting effect of 10 mg/ml insoluble glycogen (ISG) on the endotoxin (ET) shock of rats. Data calculated on the basis of three separate experiments

| Group of animals (number of rats in a group) | | Lethality (number of dead animals) |
|---|---|---|
| 1. ISG + Pb acetate | (7) | 0 |
| 2. Pb acetate + ET ($LD_{50}$) | (12) | 6 |
| 3. ISG + Pb acetate + ET ($LD_{50}$) | (12) | 0 |

**Table 2**. Binding of $^{99m}$Tc *E. coli* lipopolysaccharide (endotoxin, ET) by 1 mg of insoluble glycogen (ISG). Data represent the average values of two repeated binding experiments with duplicated samples ($n = 4$)

| Solution | Experimental samples | | | |
|---|---|---|---|---|
| | 1 | 2 | 3 | 4 |
| 1 mg ISG (0.25 ml) | 0.25 | 0.25 | 0.25 | 0.25 |
| $^{99m}$Tc ET (ml) | 0 | 0.20 | 0.40 | 0.80 |
| PBS(ml) | 1.00 | 0.80 | 0.60 | 0.20 |
| | | 37°C 60 min centrifugation | | |
| Pellet (cpm) | 33 | 45100 (23%) | 126653 (36%) | 285177 (44%) |
| Supernatant (cpm) | 45 | 149678 (77%) | 224151 (64%) | 352028 (56%) |

**Table 3**. Binding of molecules by 1 mg of insoluble glycogen (ISG). Data represent the average values of two repeated binding experiments with duplicated samples ($n = 4$)

| Types of molecules | Mass of bound molecules (mg) |
|---|---|
| Phosphorylase-a | 0.1 |
| IgG | 0.08 |
| C3b | 0.04 |
| Human secretable phospholipase $A_2$ | 0.015 |
| Tumour necrosis factor alpha | 0.003 |
| Interleukin 1 | 0.002 |

**Table 4**. Effect of insoluble glycogen (ISG) on the luminol amplified chemiluminescence of neutrophils in whole human and rat bloods. The results represent the mean values ± SD of three separate experiments wtih duplicated samples ($n = 6$)

| Samples | Chemiluminescence Number of emitted photons cpm mean ± SD ($n = 6$) |
|---|---|
| Non-stimulated blood (rat) | 816 ± 65 |
| Blood + zymosan (1 mg/ml) (rat) | 2410 ± 133 |
| Blood + ISG (1 mg/ml) (rat) | 2250 ± 107 |
| Non-stimulated blood (human) | 2020 ± 820 |
| Blood + zymosan (1 mg/ml) (human) | 7400 ± 1410 |
| Blood + ISG (1 mg/ml) (human) | 7250 ± 980 |
| Blood (human) + phosphorylase-a adsorbed to ISG (1 mg/ml) | 25400 ± 8520* |

*$P < 0.001$.

complex was added to the cells together, chemiluminescence elevated significantly ($P < 0{\cdot}001$) suggesting that the level of intracellular glucose had been elevated on the influence of phosphorylase-a, metabolizing glycogen. These observations proved functionally (we have also unpublished morphological evidences) that the particles of ISG could be internalized by phagocytes, and the intracellular glucose produced could increase the free radical production,[6] and it could serve also as a source of energy for the phagocytosing cells. These data show that cells can tolerate insoluble glycogen having dual effects: transporting toxic materials to the phagocytes,[1] and producing intracellular glucose for the cells.[2]

### Discussion

Insoluble glycogen represents a new molecular and theoretical approach for the solution of the therapeutic problems of endotoxin or septic shock. In these pathologic states, like in other forms of systemic inflammatory response syndrome (SIRS), the simultaneous presence of numerous toxic substances is the main reason for the difficulties.[1] Insoluble glycogen partly follows the principle published previously concerning the beneficial effects of adsorbents, kaolin or silicates, used endogenously in experimental intestinal infections. These materials, however, are themselves toxic.[4] Insoluble glycogen is also a potent adsorbent with the capability to adsorb, to bind simultaneously many kinds of molecules in plasma, toxic or non-toxic, as well. The opsonization of ISG by C3b and IgG has still an important advantage in plasma. These molecules enable insoluble glycogen to penetrate into the phagocytes via CR1 and Fc receptors transporting some quantities of TNF-α, IL-1, $PLA_2$, PAF, LPS, etc. into the lysosomes of phagocytes simultaneously. The special advantage of insoluble glycogen is manifested in its capability to improve the metabolism of phagocytes in toxic states. The effects of triglycerid-rich lipoproteins in preventing septic death, their influences on the transport and metabolism of endotoxin or cytokines suggest similar mechanisms as in the case of ISG.[15]

We have to mention that the protecting effect of ISG on the lethality of endotoxin shock of rats could be observed only when we injected it just before plumbum acetate and LPS. There was no beneficial effect of ISG applied in the 4th hour of shock. This finding suggests that the efficacy of an adsorbent therapy can be strongly related to the time of application and to the relative amount of toxic agents adsorbed in the acute forms of toxic shock.[16]

In the *in vitro* binding experiments for the various substances the sedimentation of ISG particles caused a technical problem during incubation. This was why we measured the binding constants using the same amount of ISG and we tested the gradually elevating concentrations of the materials. From these studies it has been apparent that ISG, as a natural substrate of phosphorylase-a, can bind this enzyme to the greatest extent. Besides, there are great differences in the binding capacities of ISG to the various substances. From the aspect of therapeutical effects, we emphasize that ISG can bind C3b, IgG, endotoxin, $PLA_2$, TNF-$\alpha$ and IL-1 simultaneously.

According to our unpublished data, ISG applied i.v. in a concentration of 10 mg/kg did not result in a significant change in the level of blood glucose. Thus, no blood sugar elevating component can be expected to take part in its protecting effect in endotoxin shock, where the fall of blood sugar is regarded as a factor of lethality.[17] The beneficial influence of ISG on the glucose metabolism can be basically related to the peripheral blood cells, mainly to phagocytes. ISG was able to increase the free radical production in the peripheral granulocytes suggesting that its particles could be readily internalized and they could be metabolized to glucose intracellularly, elevating the capability of cells for increased production of free radicals.[6] Recently we have found that ISG has a protective effect in the experimental necrotizing pancreatitis of dogs.[18]

Insoluble glycogen is representative of a family of potentially new drugs with multiple functions, serving special pharmacological purposes like detoxification, transportation, etc., besides directly helping the metabolism of some types of cells. For such an aim, the chemical modification of various natural, not toxic materials, mainly of carbohydrates or polynucleotides, can give further examples in the future, effecting as 'metabolizable internal adsorbents'.

### References

1. Doebber TW, Wu MS, Robbins JC, Choy BM, Chang MN, Sheu TY. Platelet activating factor (PAF) involvement in endotoxin induced hypotension in rats. Studies with PAF receptor antagonists kadsurenone. *Biochem Biophys Res Commun* 1985; **127**: 799–808.
2. Eskandari MK, Bolgos G, Miller C, Nguyen DT, de Forge LE, Remick DG. Anti-tumour necrosis factor antibody therapy fails to prevent lethality after cecal ligation and puncture or endotoxemia. *J Immunol* 1992; **148**: 2724–2730.
3. Farkas I, Toth P, Gergely P, Bot G. Insoluble glycogen and its interaction with phosphorylase. A novel method for the purification of liver phosphorylase-a. *Acta Biochim Biophys Hung* 1987; **22**: 17–29.
4. Gardiner KR, Anderson NH, McCaigue MD, Erwin PJ, Halliday MI, Rowlands BJ. Adsorbents as antiendotoxin agents in experimental colitis. *Gut* 1993 **34**: 51–55.
5. Hewett JA, Roth RA. Hepatic and extrahepatic pathobiology of bacterial lipopolysaccharides. *Pharmacol Rev* 1993; **45**: 381–441.
6. Hurst NP. Molecular basis of activation and regulation of the phagocyte respiratory burst. *Ann Rheum Dis* 1987; **46**: 265–272.
7. Kodoma M, Hanasawa K, Tani T. New therapeutic method against septic shock-removal of endotoxin using extracorporal circulation. *Adv Exp Med Biol* 1990; **256**: 653–661.
8. Kolltai M, Guinot P, Hosford D, Braquet PG. Platelet-activating factor antagonists: scientific background and possible clinical applications. *Adv Pharmacol* 1994; **28**: 81–145.
9. Mathison JC, Wolfson E, Ulevitch RJ. Participation of tumor necrosis factor in the mediation of Gram negative bacterial lipopolysaccharide induced injury in rabbits. *J Clin Invest* 1988; **81**: 1925–1937.
10. Mitzner S, Schneidewind J, Falkenhagen D, Loth F, Klinkmann F. Extracorporeal endotoxin removal by immobilised polyethylenamine. *Artificial Organs* 1993; **17**: 775–781.
11. Ohlson K, Bjork P, Bergenfeldt M, Hageman R, Thomson RC. Interleukin-1 receptor antagonists reduces mortality from endotoxin shock. *Nature* 1990; **348**: 550–552.
12. Selye H, Tuchweber B, Bertók L. Effect of lead acetate on the susceptibility of rats to bacterial endotoxins. *J Bacteriol* 1966; **91**: 884–890.
13. Sipka S, Szentmiklósi J, Csongor J, Taskov V, Nagy A, Szegedi Gy. Inhibition of zymosan-induced chemiluminescence of human phagocytes by adenosine, polyadenylic acid and agents influencing adenosine metabolism. *Allergol Immunopathol* 1989; **17**: 209–212.
14. Vadas P, Stefanski E, Pruzanski W. Potential therapeutic efficacy of inhibitors of human phospholipase $A_2$ in septic shock. *Agents Actions* 1986; **19**: 194–202.
15. Read TE, Grundfeld C, Kumwenda ZL, *et al.* Triglyceride-rich lipoproteins prevent septic death in rats. *J Exp Med* 1995; **182**: 267–270.
16. Bauer C, Roth W, Bahrami S, Marzi I. Attenuation of shock-induced inflammation in the rat liver depends on the time of TNF-$\alpha$ inhibition. *J Mol Med* 1996; **74**: 51–58.
17. Casado M, Díaz-Guerra MJM, Boscá L, Martín-Sanz P. Characterization of nitric oxide dependent changes in carbohydrate hepatic metabolism during septic shock. *Life Sci* 1996; **58**: 561–572.
18. Sipka S, Sápy P, Bot Gy, *et al.* Protecting effects of intravenous insoluble glycogen treatment on the experimental necrotizing acute pancreatitis of dogs. *Hepato-Gastroenterology* 1997; **44**: 127–132.

ACKNOWLEDGEMENTS. We would like to thank Dr Borbala Spett for labelling of endotoxin and Sándor Komáromi for his help in the experiments on the rats. This work was supported by National Foundation for Scientific Research, grant OTKA 1451 and Hungarian Ministry of Health and Social Welfare T-516 (1990 T-10).

**Received 13 June 1997; accepted in revised form 19 August 1997**
